# γ-Hydroxybutyric Acid (GHB) Is Not an Agonist of Extrasynaptic GABA_A_ Receptors

**DOI:** 10.1371/journal.pone.0079062

**Published:** 2013-11-11

**Authors:** William M. Connelly, Adam C. Errington, Vincenzo Crunelli

**Affiliations:** Neuroscience Division, Cardiff School of Biosciences, Cardiff University, Cardiff, United Kingdom; University Paris 6, France

## Abstract

γ-Hydroxybutyric acid (GHB) is an endogenous compound and a drug used clinically to treat the symptoms of narcolepsy. GHB is known to be an agonist of GABA_B_ receptors with millimolar affinity, but also binds with much higher affinity to another site, known as the GHB receptor. While a body of evidence has shown that GHB does not bind to GABA_A_ receptors widely, recent evidence has suggested that the GHB receptor is in fact on extrasynaptic α4β1δ GABA_A_ receptors, where GHB acts as an agonist with an EC_50_ of 140 nM. We investigated three neuronal cell types that express a tonic GABA_A_ receptor current mediated by extrasynaptic receptors: ventrobasal (VB) thalamic neurons, dentate gyrus granule cells and striatal medium spiny neurons. Using whole-cell voltage clamp in brain slices, we found no evidence that GHB (10 µM) induced any GABA_A_ receptor mediated current in these cell types, nor that it modulated inhibitory synaptic currents. Furthermore, a high concentration of GHB (3 mM) was able to produce a GABA_B_ receptor mediated current, but did not induce any other currents. These results suggest either that GHB is not a high affinity agonist at native α4β1δ receptors, or that these receptors do not exist in classical areas associated with extrasynaptic currents.

## Introduction

γ-Hydroxybutyric acid (GHB) is a naturally occurring GABA metabolite, a recreational drug and is used therapeutically as a treatment for the symptoms of narcolepsy and to treat alcohol dependence and withdrawal [1]. GHB causes dose-dependent sedation, ataxia and hypothermia, however, the molecular mechanism of action is unclear. GHB binds with low micromolar affinity to GABA_B_ receptors and many of its effects can be directly ascribed to activation of these receptors. Specifically, pretreatment with specific GABA_B_ receptor antagonists prevents GHB-induced hypothermia, ataxia and sedation, effects which GABA_B(1)_^−/−^ mice are also resistant to [2]–[5]. In vitro, GHB has been shown to hyperpolarize neurons by opening potassium channels and decrease neurotransmitter release, by inhibiting calcium channels, again an effect that is generally reported to be mediated specifically by GABA_B_ receptors [6]–[8]. GHB also binds to at least one other receptor apart from the GABA_B_ receptor, the so-called “GHB receptors”. GHB binds to this site with nano- to micromolar affinity and is antagonised by the compound NCS-382. In apparent disagreement with the work already cited, NCS-382 has been demonstrated to block GHB-induced hypolocomotion and ataxia [9][though see 2]. Similarly, the purported specific GHB receptor agonist γ-hydroxyvaleric acid mimics some of the effects of GHB (e.g. ataxia, sedation) without binding to the GABA_B_ receptor [10]. Likewise, one report shows that GHB reduces GABA release via a presynaptic mechanism that is blocked by NCS-382 [11]. While a high affinity target of GHB has been cloned, it is not sensitive to NCS-382 [12]. Therefore, the exact molecular nature of the GHB receptor is unclear.

Several studies have shown that GHB does not directly affect the function of cortical synaptic GABA_A_ receptors [7], [13], [14][though see 15]. Likewise, there are several reports showing that neither GHB nor NCS-382 affect the binding of classical GABA_A_ receptor ligands [16]–[18][though see 19]. However, a recent study by Absalom et al., (2012) concluded that GHB binds to extrasynaptic GABA_A_ receptors [20]. Extrasynaptic GABA_A_ receptors are classically high-affinity α4βδ or α6βδ receptors expressed in the perisynaptic or extrasynaptic space, which mediate a tonic inhibitory current [21]. These receptors are found in the highest density in dentate gyrus granule cells, the ventrobasal (VB) thalamic neurons and cerebellar granule cells, but they can also be found in striatal medium spiny neurons and to a lesser extent in neocortical pyramidal cells [22]. Using a Xenopus oocytes expression system, Absalom et al., (2012) demonstrated that GHB was a high potency partial agonist of α4β1δ GABA_A_ receptors [EC50 = 140 nM], but only activated α4β2/3δ GABA_A_ receptors in the millimolar range. Furthermore, they showed that this effect was absolutely dependent on α4 and δ subunits and was blocked by classical antagonists of the GABA_A_ receptor. In native tissue, they went on to show that the B_max_ of [3H]NCS-382 binding was significantly reduced in α4 subunit knockout mice (though not in δ subunit knock outs).

Therefore, we sought to investigate where GHB was a functional agonist of extrasynaptic α4βδ receptors in the rat brain. We elected to examine this in VB thalamocortical neurons, dentate gyrus granule cells and striatal medium spiny neurons, as all these cell types express a measurable tonic current mediated by α4βδ neurons and are believed to express the β1 subunit (though generally in lower abundance than β2 or β3) [22]–[25].

## Materials and Methods

All experiments were carried out in accordance with the United Kingdom Animals (Scientific Procedures) Act 1986 and with local ethical committee (Cardiff University Research Ethics Committee) approval. All efforts were made to minimize animal suffering and keep the animal numbers to a minimum. Animals used were Wistar rats of either sex (postnatal days 20–30), feed ad libitum and kept on a 12 h light/dark cycle.

Animals were anesthetised with 5% isoflurane/95% O_2_ and rapidly decapitated. The brain was dissected out into 4°C sucrose artificial cerebrospinal fluid (aCSF) bubbled with 95% O_2_/5% CO_2_ of the following composition (in mM) 85 NaCl, 60 sucrose 2.5 KCl, 25 NaHCO_3_, 1.25 NaH_2_PO_4_, 2 MgCl_2_, 1 CaCl_2_, 25 D-glucose, 3 kynurenic acid. The brain was cut using a vibrotome (Microm HM 650 V, Thermo Fisher Scientific) into 300 µm horizontal sections of the VB thalamus and dentate gyrus, or coronal sections for the dorsal striatum. Slices were transferred to a holding chamber filled with sucrose aCSF where they were maintained at 35°C for half an hour before being allowed to cool to room temperature. At that time, the sucrose aCSF was slowly exchanged with aCSF of the following composition (in mM) 125 NaCl, 2.5 KCl, 25 NaHCO_3_, 1.25 NaH_2_PO_4_, 2 MgCl_2_, 1 CaCl_2_, 25 D-glucose. After a further half hour, slices were transferred to the recording chamber as needed, and kept for a maximum of 8 hours.

Brain slices were perfused with aCSF of the following composition at 2–3 mL min^−1^ (in mM) 125 NaCl, 2.5 KCl, 25 NaHCO_3_, 1.25 NaH_2_PO_4_, 1 MgCl_2_, 2 CaCl_2_, 25 D-glucose which was maintained at ∼34°C (TC2-BIP, Cell Microcontrols, USA). CNQX (10 µM) and D-AP5 (25 µM) were added to the recording solution at all times to block glutamatergic currents. Neurons were visualized using either a Nikon Eclipse E600FN (Tokyo, Japan) or Olympus BX51 (Tokyo, Japan) microscope equipped with a 40 or 60× immersion lens and a video camera (Hamamatsu, Hamamatsu City, Japan). To investigate whether GHB (100 µM) or THIP (1 µM) enhanced the tonic current or altered synaptic events whole-cell patch-clamp recordings were made from neurons held at –70 mV using pipettes (resistance, 2–4 MΩ) containing the following (in mM): 130 CsCl, 4 Mg-ATP, 0.3 Na-GTP, 10 Na-HEPES, and 0.1 EGTA, pH 7.25 (osmolality, ∼295 mOsm). When GHB (3 mM) was used, to allow the GABA_B_ receptor mediated effects to be recorded, the pipettes were filled with the following (in mM): 130 K-MeSO_4_, 5 NaCl, 4 Mg-ATP, 0.3 Na-GTP, 10 Na-HEPES, and 0.1 EGTA, pH 7.25 (osmolality, ∼295 mOsm) at the cells were held at −50 mV. Experimental data was filtered at 3–6 kHz, digitized at 20 kHz (Digidata 1322A; Axon Instruments) and acquired using pClamp 10 software (Axon Instruments). Series resistance (R_s_) was measured at the beginning of the recording, and if it was over 20 MΩ the experiment was ended. R_s_ was measured again at the end of the recording and if it had changed by more than 20% since the experiment had begun, the data was discarded. For VB neurons the mean initial R_s_ was 14.4±0.8 MΩ and this had increased to 15.7±0.8 MΩ (n = 22) by the end of the recording session. For dentate gyrus granule cells the mean R_s_ value was 9.5±1 MΩ which increased to 10.3±1.2 MΩ (n = 17) by the end of the experiment. For striatal neurons the initial R_s_ was 10.7±0.5 MΩ and by the end of the experiment this was 11.4±0.7 MΩ (n = 23). Nucleated patches were pulled using standard methods and the CsCl based pipette solution described above and held at −70 mV [26]. Nucleated patches were acceptable if they were roughly spherical, had a whole-cell capacitance of <3 pF and an input resistance of >500 MΩ. Drugs were puff applied onto patches for 2 seconds every 20 seconds using a custom built TTL-driven picospritzer system from a pipette with a 5–10 µm tip held ∼50 µm from the patch. Drugs were dissolved in standard aCSF. To allow representative images of cells to be imaged and classical membrane properties to be recorded, occasionally 25 µM Alexa Fluor 594 was added to K-MeSO_4_ pipette solution. Two-photon laser-scanning microscopy (2P-LSM) was performed using a Prairie Ultima (Prairie Technologies) microscope powered by a titanium:sapphire pulsed laser (Chameleon Ultra II; Coherent) tuned to λ = 810 nm. Maximum intensity projections were constructed from Z series of images taken with 1 µm focal steps using MetaMorph software (Molecular Devices) and ImageJ (NIH).

GHB was purchased from Sigma-Aldrich. All compounds used for aCSF and pipette solution were purchased from Fischer Scientific or Sigma-Aldrich. CNQX, D-AP5, CGP 55845, Tetrodotoxin (TTX) and 4,5,6,7-tetrahydroisoxazolo[5,4-c]pyridin-3-ol (THIP) were purchased from Tocris.

To measure the holding current in a way that was not affected by spontaneous IPSCs, an all points sample of the data was collected every second, and the mode of this sample was used to define the holding current. To measure the effect of a drug on the holding current an average of 10 seconds of these samples just prior to the application of the drug was used to create the control value, and 10 seconds of samples at the end of drug application was used to create the drug value. Miniature IPSCs were detected using the template function of Axograph X (Axograph Scientific). Drug induced changes in holding current and drug-puff induced currents were tested with paired *t*-tests and one sample *t*-tests respectively, using Graphpad Prism. mIPSC amplitude distributions were tested with Kolmogorov-Smirnov tests using pClamp. *P*<0.05 was taken as significant.

## Results

**Figure 1:**
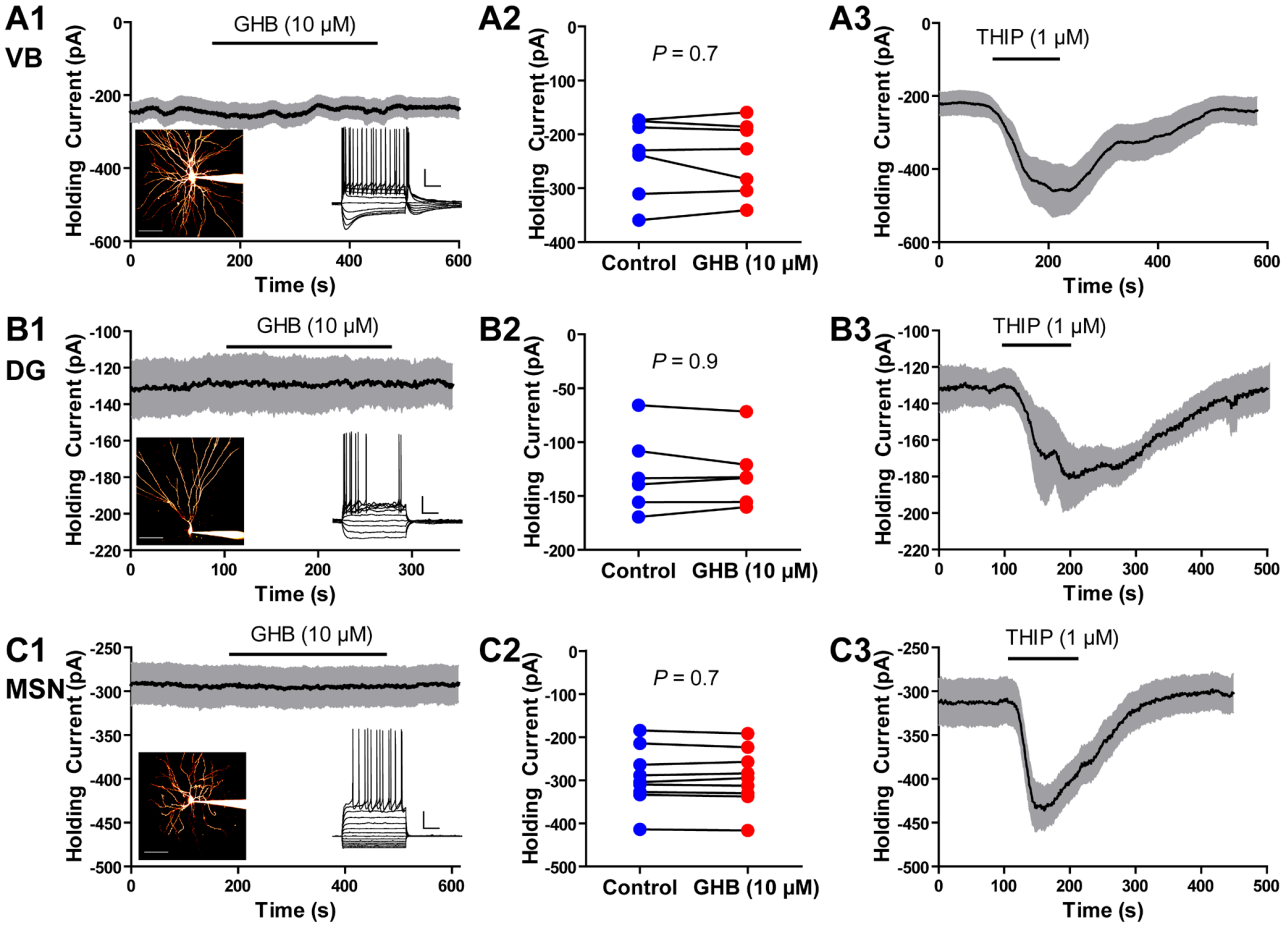
GHB has no effect on the holding current of voltage clamped neurons. ***A1***, Mean holding current over time in response to the application of GHB in VB thalamic neurons (n = 6). Grey area shows SEM. Left inset, representative image of a VB neuron. Scale bar 50 µm. Right inset, voltage response of a representative VB neuron to current injection. Scale bar 20 mV 200 ms. ***A2***, Group results showing the mean holding current before and after GHB. ***A3***, The δ-selective GABA_A_ agonist THIP induced a significant inward current (control: −221±33 pA, THIP: −508±53 pA, n = 6, *P* = 0.0007). ***B1***, Mean holding current over time in response to the application of GHB in dentate gyrus (DG) granule cell (n = 6). Grey area shows SEM. Left inset, representative image of a dentate gyrus granule cell. Scale bar 50 µm. Right inset, voltage response of a representative dentate gyrus granule cell to current injection. Scale bar 20 mV 200 ms. ***B2***, Group results showing the mean holding current before and after GHB. ***B3***, THIP induced a significant inward current (control: −131±10 pA THIP: −211±22 pA, n = 7, *P* = 0.01). ***C1,*** Mean holding current over time in response to the application of GHB in striatal medium spiny neurons (MSN) (n = 9). Grey area shows SEM. Left inset, representative image of a medium spiny neuron. Scale bar 50 µm. Right inset, voltage response of a representative medium spiny neuron to current injection. Scale bar 20 mV 200 ms. ***C2***, Group results showing the mean holding current before and after GHB. ***C3***, THIP induced a significant inward current (control: −314±26 pA THIP: −457±26 pA, n = 10, *P*<0.0001).

To investigate whether GHB activated α4βδ receptors we first performed whole cell voltage clamp recordings from VB thalamic neurons using a CsCl based internal solution. Under these conditions ([Cl_i_] = 130 mM, [Cl_o_] = 133.5 mM, V_hold_ = −70 mV), if GHB activated any significant population of GABA_A_ receptors we would expect to see an inward current. We applied GHB at 10 µM because this is approximately 100 times the EC_50_ concentration for activating α4β1δ receptors but approximately 100 times less than its EC_50_ for activating GABA_B_ receptors or α4β2/3δ receptors [20], [27]. GHB at this concentration had no effect on the holding current (control: −239±27 pA, GHB: −242±26 pA; n = 7, *P* = 0.75; Fig. 1A1–2). To confirm the validity of our assay, we applied the δ selective agonist THIP (1 µM) [28] and this produced a significant inward current (n = 6, *P* = 0.007; Fig. 1A3). We repeated this experiment in dentate gyrus granule cells and again saw that GHB (10 µM) had no effect on the holding current (control: −129±15 pA, GHB −129±13 pA; n = 6, *P* = 0.91; Fig. 1B1–2). Again, THIP induced a significant inward current (n = 7, *P* = 0.01). Finally, we patched striatal cells. With our CsCl based patch solutions, we could not confirm whether all cells we recorded from were medium spiny neurons, however, we targeted neurons with small somata (diameter <15 µm) making it unlikely that we accidentally recorded from cholinergic interneurons. Likewise, the fact that medium spiny neurons make up approximately 95% of all striatal cells, makes it further unlikely that we recorded from other cell types [29]. However, again, GHB (10 µM) had no effect on the holding current (control: −293±23 pA, GHB: −294±22 pA; P = 0.7, n = 9; Fig. 1C1–2), while THIP produced a significant inward current (n = 10, *P*<0.0001).

**Figure 2:**
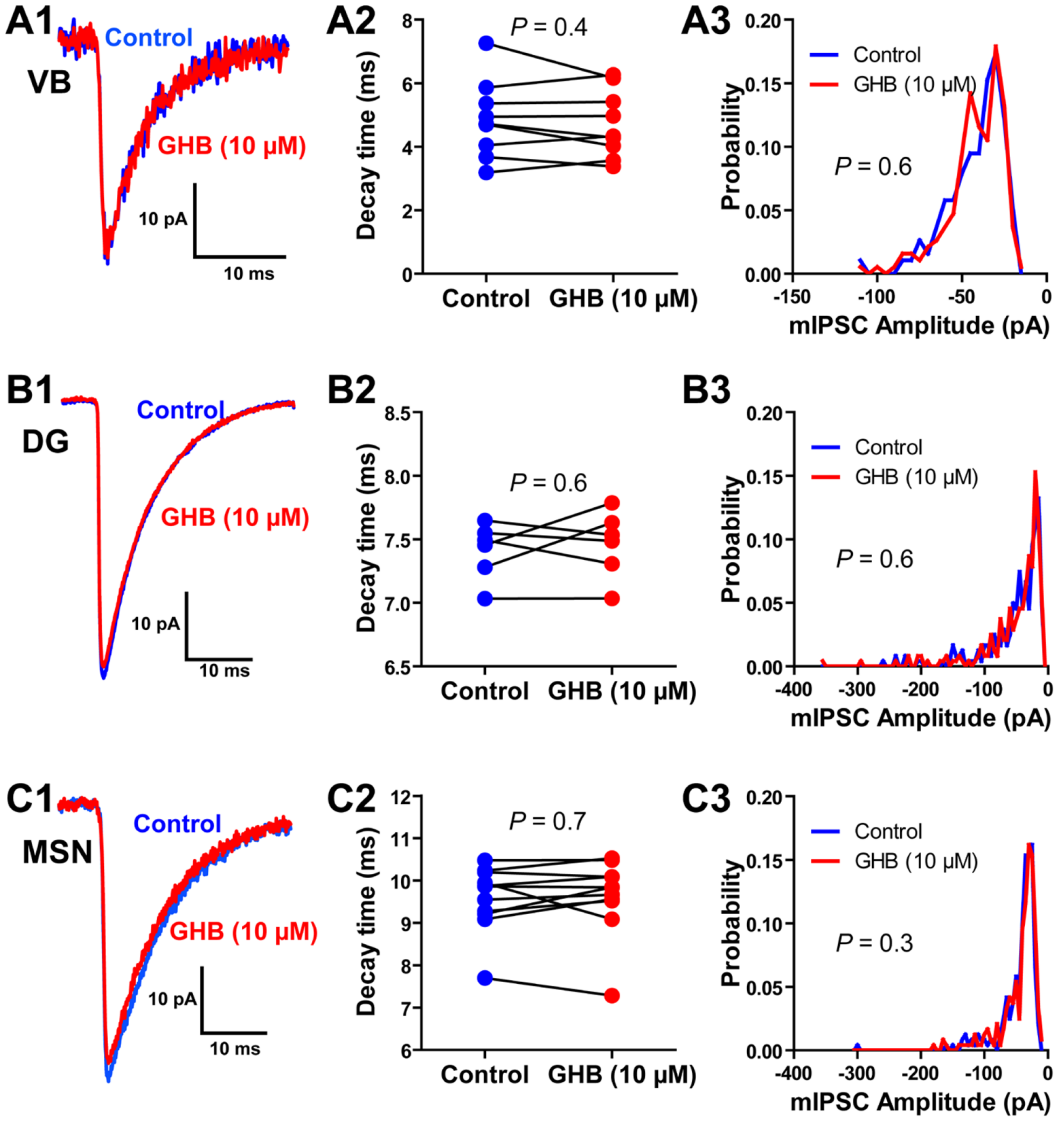
GHB has no effect on synaptic GABA_A_ receptors. ***A1***, Representative average mIPSC from a VB thalamic neuron before and after application of GHB. ***A2***, GHB has no effect on the decay time constants of averaged mIPSCs in VB neurons. ***A3***, Distribution of mIPSCs amplitude across 9 cells, showing no effect of GHB. ***B1***, Representative average mIPSC from a dentate gyrus (DG) granule cell before and after application of GHB. ***B2***, GHB has no effect on the decay time constants of averaged mIPSCs in dentate gyrus granule cells. ***B3***, Distribution of mIPSCs amplitude across 6 cells, showing no effect of GHB. ***C1***, Representative average mIPSC from a striatal medium spiny neuron (MSN) before and after application of GHB. ***C2***, GHB has no effect on the decay time constants of averaged mIPSCs in medium spiny neurons. ***C3***, Distribution of mIPSCs amplitude across 11 cells, showing no effect of GHB.

These results seem to indicated that GHB (10 µM) does not modulate extrasynaptic receptors in VB thalamic neurons, dentate gyrus granule cells and striatal medium spiny neurons. However, because the exact subunit composition of synaptic and extrasynaptic receptors is unclear (especially when it comes to which β subunits are involved), and hence there is a chance that GHB sensitive GABA_A_ receptors are expressed synaptically, we sought to confirm whether GHB modulates synaptic GABA_A_ receptor mediated currents. Miniature IPSCs (mIPSCs) were isolated with TTX (500 nM). In the VB thalamus, mIPSCs had a mean amplitude of 40±4 pA and a mean decay time constant of 4.9±0.4 ms, however GHB (10 µM) had no effect on these parameters (36±4 pA, 4.7±0.4 ms; n = 9, *P* = 0.5, *P* = 0.4 respectively; Fig. 2A). Furthermore, GHB (10 µM) had no effect on the distribution of mIPSC amplitude (*P* = 0.6; Fig. 2A3). Similarly, GHB (10 µM) had no effect on mIPSCs recorded in dentate gyrus granule cells (control: 42±2 pA, 7.4±0.1; GHB: 41±2 pA, 7.5±0.1 ms; n = 6, *P* = 0.5, *P* = 0.6 respectively; Fig. 2B). Finally, GHB (10 µM) had no effect on mIPSCs recording from striatal medium spiny neurons (control: 39±4 pA, 9.6±0.2 ms; GHB: 39±4 pA, 9.6±0.3 ms; n = 11, *P* = 0.8 *P* = 0.7 respectively; Fig. 2C).

**Figure 3:**
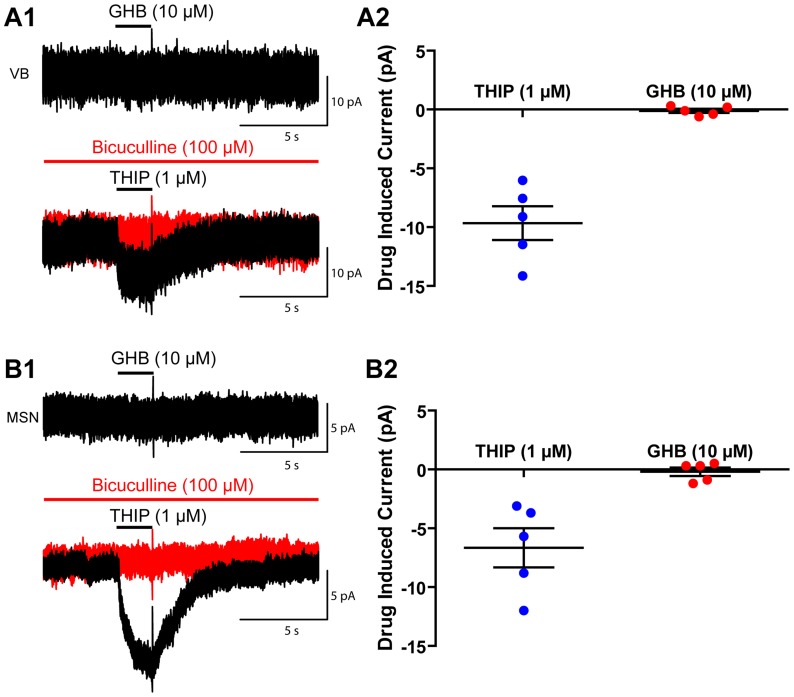
Puff application of GHB has no effect on nucleated patches pulled from VB thalamocortical cells, or medium spiny neurons. A1, Example traces showing the effect of 2 second puff applications of GHB (10 µM) and THIP (1 µM) to nucleated patches of thalamocortical VB neurons. A2, Group results showing the magnitude of the effect of THIP and GHB in VB neurons. B1, Example trace showing the effect of 2 second puff application of GHB (10 µM) and THIP (1 µM) to nucleated patches of medium spiny neurons. A2, Group results showing the magnitude of the effect of THIP and GHB in medium spiny neurons.

Endogenous GHB is found in the CSF at approximately 100 nM (and >1 µM in bulk tissue), and the concentration is reported to increase after death [30], [31], therefore it seems possible that endogenous GHB is saturating any high affinity GHB receptors in our brain slices. Therefore, we sought to avoid this problem by puff applying GHB onto nucleated patches drawn above the surface of the slice (>200 µm). We avoided using dissociated cell culture, due to the risk of ectopic expression of ion channels not expressed in native tissue. Likewise, we did not use outside-out patches, as that would mean we could only sample a small area of membrane, potentially missing a receptor expressed at low density. Unfortunately, nucleated patches only allow us to sample the somatic membrane. As it is known that the α4βδ expressed by dentate gyrus granule cells are restricted to the distal dendrites, we excluded using these cells for this experiment [32]. Puff application of THIP (1 µM) demonstrated a small bicuculline-sensitive current in both thalamocortical (−9.7±1.4 pA, n = 5, *P* = 0.002) and medium spiny neurons (−6.6±1.7 pA, n = 5, *P* = 0.01) (Fig. 3). However, again, low concentrations of GHB (10 µM), induced no significant current. (VB: −0.1±0.2 pA, n = 5, *P* = 0.5; medium spiny neurons: −0.2±0.4, n = 5, *P* = 0.6) (Fig. 3).

**Figure 4:**
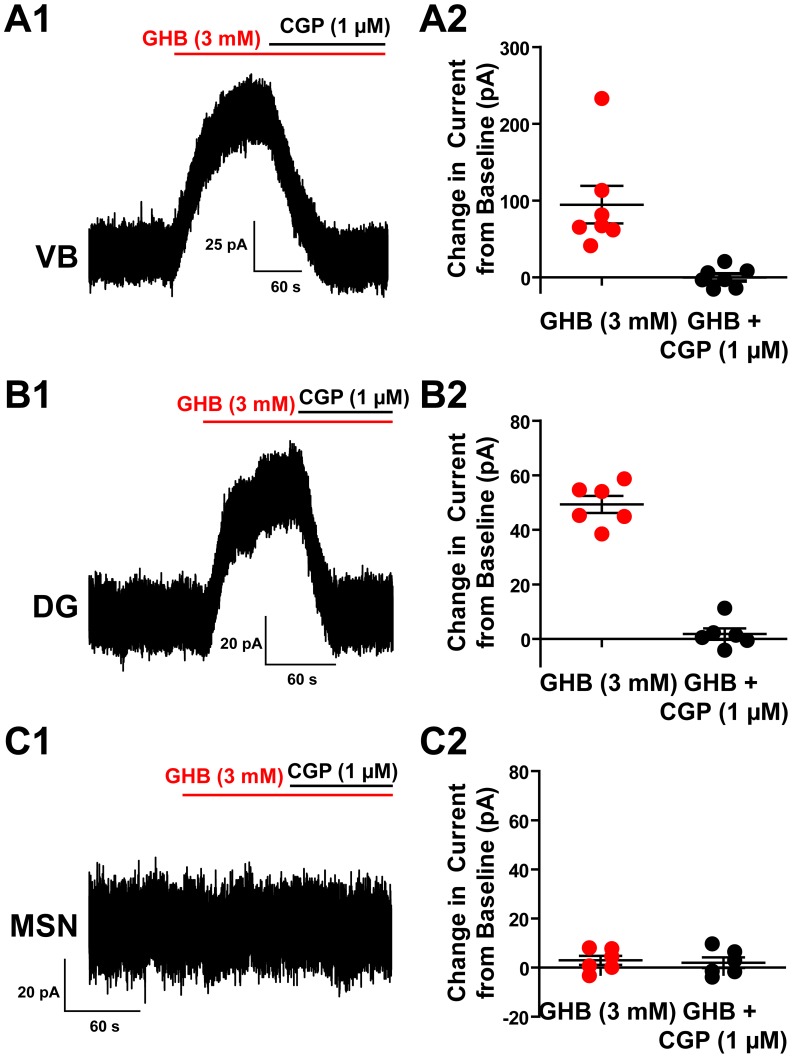
High concentrations of GHB can induce an inward current that is completely abolished by the GABA_B_ antagonist GCP 55845. ***A1***, Representative recording from a VB thalamic neuron showing the inward current induced by 3 mM GHB, and its blockade by CGP 55845 revealing no residual current. ***A2***, Group results showing the current induced by GHB and its complete blockade by CGP 55845. ***B1***, Representative recording from a dentate gyrus (DG) granule cells showing the inward current induced by 3 mM GHB, and its blockade by CGP 55845 revealing no residual current. ***B2***, Group results showing the current induced by GHB and its complete blockade by CGP 55845. ***C1***, Representative recording from a striatal medium spiny neuron (MSN) the complete lack of response to 3 mM GHB and CGP 5584. ***C2***, Group results showing the lack of effect of GHB or CGP 55845.

To confirm that the lack of action of GHB was not due to some alteration in the concentration dependence between expression systems and brain slices, we applied GHB at 3 mM while using a K-MeSO_4_ based pipette solution with [Cl]_i_ = 5 mM and voltage clamped the cells at −50 mV. Under these conditions chloride currents have a calculated reversal potential of −84 mV and hence would be expected to produce outward currents. When GHB (3 mM) was applied to VB thalamic neurons, a significant outward current was produced (94±24 pA; n = 7, *P* = 0.008) (Fig. 4A). However, this current was solely the result of GHB activating GABA_B_ receptors, as the outward current was abolished by co-application of the GABA_B_ antagonist CGP 55845 (1 µM) (0±5 pA; n = 7, *P* = 0.96). Likewise, in dentate gyrus granule cells, GHB (3 mM) produced a significant outward current that was completely blocked by CGP 55845 (1 µM) (GHB: 49±3 pA, CGP: 2±2 pA; n = 6, *P*<0.0001, *P* = 0.4 respectively) (Fig. 4B). However, in medium spiny neurons, GHB (3 mM) did not produce any significant current and CGP 55845 (1 µM) had no further effect (GHB 3±2 pA, CGP: 2±2 pA; n = 6, *P* = 0.2, *P* = 0.4 respectively) (Fig. 4C). This is in line with previous reports that the GABA_B_ receptor has no post-synaptic effect on medium spiny neurons [33].

## Discussion

It is well established that GHB is an agonist at GABA_B_ receptors, however, evidence suggests that it also has some behavioural effects via another site: the GHB receptor. For instance, NCS-382, the GHB receptor antagonist has been demonstrated to block GHB-induced hypolocomotion and ataxia [9]. A recent study by Absalom et al., (2012) suggests that the GHB receptor is in fact the α4β1δ GABA_A_ receptor, and that GHB is a direct agonist at these receptors [20]. We recorded from three cell types that express the mRNA for these subunits, and express a GABA_A_ mediated current with the pharmacological hall marks of α4βxδ receptors: VB thalamic neurons, dentate gyrus granule cells and striatal MSNs [23], [34]–[38]. In these cell types we found no evidence that 10 µM GHB affected synaptic or extrasynaptic GABA_A_ receptors, nor that it evoked a measureable current when applied to nucleated patches. Furthermore while 3 mM GHB activated GABA_B_ receptors, there was no indication that it activated any GABA_A_ receptors.

There are at least three potential explanations for the differences seen between our results and those of Absolom et al., (2012). The first is that their results are due to an artefact of the heterologous expression system used. This seems unlikely as their autoradiographic experiments show clear GHB and NCS-382 binding that is reduced in α4−/− mice. The second possibility is that the α4β1δ receptor is expressed by only a few cells in the regions we sampled from. This is always an issue with single cell experiments. However, if the cells are sufficiently rare that in a sample of 77 neurons/patches to which GHB was applied, not a single sign of a GABAA receptor mediated current was seen, it calls into question the functional significance of this GHB at this receptor combination. The other explanation is that the regions we studied do not express α4β1δ GABA_A_ receptors at all, but instead express α4β2/3δ receptors. The pharmacological and knockout evidence makes it clear that VB thalamic neurons, dentate gyrus granule cells and striatal medium spiny neurons express α4βxδ GABA_A_ receptors [24], [25], [36]–[38], however, which β subunit is expressed with these receptors is less clear. The fact that the β2/3 selective compound etomidate enhances the tonic current in VB neurons shows that the tonic current is in part mediated by β2/3 containing GABA_A_ receptors [39]. However, given that the tonic current of β2^−/−^ mice is only reduced by approximately 50%, it indicates that the tonic current in these neurons is partially produced by β1 and/or β3 containing receptors [39]. Importantly, the tonic current from mice with the etomidate insensitive β2_N265S_ mutation is still enhanced by etomidate, showing that the tonic current is in part mediated by β3 receptors, however it is unclear whether β1 containing receptors also mediated part of the tonic current. At least at α6βxγ2 receptors, etomidate has very similar modulator properties at β2 and β3 containing receptors [40]. However, in VB neurons from β2^−/−^ mice, etomidate enhances the tonic current significantly less, suggesting that some of the tonic current is mediated by β1 containing receptors (though this could be due to a compensatory upregulation of β1 subunits in response to a loss of β2 subunits) [39]. In the dentate gyrus an identical approach shows that neurons from β2^−/−^ mice have a halved tonic current, and that the tonic currents from β2_N265S_ mice are still partially sensitive to etomidate, showing the presence of β3 containing extrasynaptic receptors. Again, the magnitude of the etomidate-induced enhancement of the tonic current in β2^−/−^ mice suggests the presence of some β1 containing receptors [41] Furthermore, a recent study has shown that protein kinase A (PKA) inhibits the current mediated by extrasynaptic receptors in VB thalamic neurons [42]. As PKA has been shown to inhibit β1 containing receptors, while having no effect on those containing β2 and enhancing β3 containing receptors, this indicates that VB thalamic neurons contain a significant amount of β1 containing GABA_A_ receptors, at least at extrasynaptic sites [43], [44]. While these results make it hard to say conclusively which β subunits are expressed at the extrasynaptic GABA_A_ receptors in these nuclei, it is worth noting that Absalom et al., (2012) did show clear gabazine sensitive [^3^H]NCS-382 binding in the thalamus, dentate gyrus and striatum, showing that this site does appear to be present in the brain regions we studied [20].

While our results show no indication that GHB is a direct agonist of GABA_A_ receptors, it is worth noting that GHB can potentiate extrasynaptic receptors, however, we have shown that this is through an indirect mechanism, relying of GABA_B_ receptors [35], [42]. These results showed that activation of the GABA_B_ receptor leads to a dephosphorylation of presumably α4β1δ containing receptors and a subsequent potentiation of extrasynaptic GABA_A_ currents [42]. However, we cannot use these results to explain the findings of Absalom et al., (2012) [20].

Given that we could see no indication that GHB was an agonist of GABA_A_ receptors in VB thalamus neurons, dentate gyrus granule cells or striatal medium spiny neurons, we are left with three possibilities, 1) that GHB is not an agonist of α4β1δ native receptors, 2) the receptors are expressed in rare cell populations or 3) that these receptors do not exist in these brain regions. We cannot rule out any of these option. However, if these receptors do exist in any significant number, we feel it is unlikely that GHB is as potent an agonist as suggested by Absolom et al., (2012) [20]. This is due to the fact that all known reports of a behavioural effect due to GHB dosing require doses of ≥100 mg/kg, even those reported to be antagonised NCS-382 [e.g. 3,9,10]. Seeing as doses of 120 mg/kg (I.V in the cat) produce cerebrospinal fluid (CSF) concentrations of ∼2.5 mM, and even endogenous GHB is found in the CSF at 100 nM (and >1 µM in bulk tissue), it would seem that the α4β1δ would be being activated at near its EC_50_ basally, and would be well past saturation before a behaviourally relevant concentration is reached [45], [31], [30]. Whether the effect of low concentrations of GHB on α4β1δ receptors is an artefact of the heterologous expression system, or simply that α4β1δ is not found in the CNS, our results call into question the relevance of the proposed direct action of GHB on extrasynaptic GABA_A_ receptors in native tissue.
